# Connaissances, attitudes et pratiques des médecins de première ligne en matière d’aide au sevrage tabagique à Sfax (Tunisie), 2020

**DOI:** 10.11604/pamj.2022.42.83.27738

**Published:** 2022-05-31

**Authors:** Maroua Trigui, Jihen Jdidi, Yosra Mejdoub, Houda Ben Ayed, Mariem Ben Hmida, Maissa Ben Jmaa, Raouf Karray, Sourour Yaich, Mondher Kassis, Habib Fki, Jamel Damak

**Affiliations:** 1 Service de Médecine Communautaire et d’Epidémiologie, Centre Hospitalier Universitaire Hèdi Chaker, Sfax, Tunisie,; 2 Service de Médecine Préventive et d´Hygiène Hospitalière, Centre Hospitalier Universitaire Hèdi Chaker, Sfax, Tunisie

**Keywords:** Médecins de première ligne, tabagisme, sevrage tabagique, First-line physicians, smoking, smoking cessation

## Abstract

**Introduction:**

le médecin de première ligne doit jouer un rôle clef dans la lutte antitabac. Les objectifs de cette étude étaient d´évaluer les connaissances, les attitudes et les pratiques des médecins de première ligne en matière d´aide au sevrage tabagique, évaluer leur statut tabagique et déterminer les obstacles auxquels ils font face dans l´aide au sevrage tabagique.

**Méthodes:**

nous avons mené une étude transversale, durant le mois de novembre 2020, auprès d´un échantillon représentatif de médecins de première ligne exerçant au niveau du gouvernorat de Sfax.

**Résultats:**

au total, 115 médecins de première ligne étaient inclus avec un sex ratio (H/F) de 0,91. L´âge médian était de 43 ans (Intervalle interquartile = [34-55 ans]). Parmi les répondants, 26 (22,6%) ont déclaré être des fumeurs. Nous avons noté que 98 médecins enquêtés (85,2%) n´ont pas eu de formation en postuniversitaire sur l´aide au sevrage tabagique. Cependant, 71 (61,7%) avaient une idée sur la thérapie de substitution nicotinique. Concernant les attitudes, 73 répondants (63,5%) étaient convaincus qu´il est de la responsabilité des médecins d´aider leurs patients à arrêter de fumer. Par rapport aux pratiques, 45 médecins (39,1%) interrogeaient systématiquement tous les patients sur leurs habitudes tabagiques. Les activités les moins exécutées de la stratégie des 5A étaient les composantes «Aider» (14%) et «Organiser un suivi» (17,4%). Le désintérêt des patients était jugé, par 61 médecins enquêtés (53%), un obstacle important pour l´aide au sevrage tabagique.

**Conclusion:**

il est nécessaire d´évaluer et d´améliorer l´application de la stratégie nationale de lutte anti-tabac, surtout en ce qui concerne la formation des médecins de première ligne.

## Abstract (en)

**Introduction:**

first-line physicians should play a key role in tobacco control. The purpose of this study is to assess the knowledge and attitudes of front-line physicians as well as their practices for smoking cessation, assess their smoking status and determine the barriers to smoking cessation support.

**Methods:**

we conducted a cross-sectional study among a representative sample of front-line physicians practising in the governorate of Sfax in November 2020.

**Results:**

a total of 115 first-line physicians were included in the study, with a sex ratio (M/F) of 0.91 and an average age of 43 years (interquartile interval= [34-55 years]). Among the respondents, 26 (22.6%) stated that they were smokers; 98 of the physicians surveyed (85.2%) had not had any postgraduate training in smoking cessation. However, 71 (61.7%) had an idea on nicotine replacement therapy. Regarding attitudes,73 respondents (63.5%) were convinced that physicians were responsible for helping their patients quit smoking. Forty five physicians (39.1%) systematically asked all patients about their smoking habits. The least performed activities of the 5A strategy were the components “help” (14%) and “organize follow-up” (17.4%). Patients’ disinterest was considered (53%) to be a significant barrier to smoking cessation assistance by the 61 physicians surveyed.

**Conclusion:**

there is a need to evaluate and improve the implementation of the national tobacco control strategy, in particular with regard to the training of front-line physicians.

## Introduction

L´épidémie de tabagisme demeure l´une des plus graves menaces ayant jamais pesé sur la santé publique mondiale puisque le tabac représente la principale cause évitable de décès dans le monde. En effet, plus de 8 millions de personnes meurent du tabagisme chaque année [1]. Le nombre de fumeurs dans le monde était estimé à 1,337 milliards en 2018 [2], dont plus de 80% vivaient dans des pays à revenu faible ou intermédiaire, là où la charge de morbidité et de mortalité liées au tabac était la plus lourde [1]. En réponse à la mondialisation de l´épidémie de tabagisme, un engagement mondial a été pris en 2003 avec l´adoption de la Convention cadre pour la lutte antitabac (CCLAT) de l´Organisation mondiale de la Santé (OMS) [1]. En Tunisie, la situation est alarmante puisque le tabagisme constitue un véritable fléau, en particulier chez les hommes, en milieu rural et chez les individus dont le niveau socio-économique et le niveau d´instruction sont faibles [3]. Selon les estimations de l´OMS en 2018, la Tunisie avait la plus grande population de fumeurs après le Liban, parmi les pays de la région de la Méditerranée orientale [2].

En effet, la prévalence du tabagisme, standardisée selon l´âge en 2018 en Tunisie, était de 26% avec une prévalence de 49,1% chez les hommes et 2,9% chez les femmes [2]. Ainsi, la mise en place d´une stratégie nationale de lutte antitabac était une des priorités des programmes de la santé publique en Tunisie [4]. Tous les personnels de santé doivent être impliqués dans la lutte antitabac, d´une part en appliquant la réglementation antitabac et d´autre part en participant à la sensibilisation et à l´aide au sevrage tabagique [4]. Il a été démontré que les fumeurs qui ont eu recours à l´aide à l´abandon du tabac avaient plus de chance de réussir leur sevrage par rapport à ceux qui n´en avaient pas [5]. En effet, seules 7% des tentatives de sevrage aboutissent sans aide [5]. A cet égard, le médecin de première ligne doit jouer un rôle clef dans la lutte antitabac, étant donné qu´il s´agit du premier relais entre la population et le système de santé. Le médecin de première ligne a l´opportunité de voir ses patients souvent et donc de répéter les messages en vue d´arrêter de fumer. Celui-ci, connaissant le statut social et économique de ses patients, leur état psychique, leurs problèmes familiaux et leurs autres dépendances, peut adapter le type d´intervention aux spécificités de chaque fumeur. Dans cette perspective, nous avons mené ce travail ayant pour objectifs d´évaluer les connaissances, les attitudes et les pratiques des médecins de première ligne en matière d´aide au sevrage tabagique, évaluer leur statut tabagique et déterminer les obstacles auxquels ils font face dans l´aide au sevrage tabagique.

## Méthodes

**Type de l´étude et population étudiée:** nous avons mené une étude transversale, durant le mois de novembre 2020, auprès d´un échantillon représentatif de médecins de première ligne exerçant au niveau du gouvernorat de Sfax. Nous avons inclus les médecins de première ligne qui ont été sélectionnés à l´échantillonnage et qui ont accepté de participer à l´étude, faisant des consultations de médecine générale dans des centres de santé de base ou dans leurs cabinets privés. Nous n´avons pas inclus dans l´étude les médecins de première ligne retraités. Nous avons exclu les médecins ayant refusé de participer à l´enquête. La liste des médecins de première ligne exerçants à Sfax a été obtenue auprès du conseil régional de l´ordre des médecins. Les médecins ont ensuite été stratifiés par secteur d´activité : public ou privé. Une allocation proportionnelle, basée sur le nombre de médecins dans chaque secteur, a été utilisée pour déterminer la proportion de médecins à échantillonner dans chaque secteur. Les médecins de chaque strate ont ensuite été sélectionnés à l´aide d´une technique d´échantillonnage aléatoire simple. La variable d´intérêt principal était la proportion des médecins qui demandent toujours le statut tabagique des patients. Pour déterminer la prévalence de la variable d´intérêt, nous nous étions basés sur les résultats d´une étude menée en Égypte et qui a trouvé une prévalence égale à 60% [6]. Ainsi, le nombre minimal de sujets nécessaires pour notre étude était de 115 médecins pour une précision de 9%.

**Recueil des données:** les données ont été recueillies à l´aide d´auto-questionnaires, rédigés en français, comportant cinq sections à savoir: les caractéristiques sociodémographiques des médecins, les connaissances et les formations des médecins sur les outils d´aide au sevrage tabagique, l´attitude des médecins à l´égard de l´aide au sevrage tabagique, les pratiques des médecins, en matière d´aide à l´arrêt du tabagisme, basées sur la stratégie des cinq «A» (demander, conseiller, évaluer, aider et organiser le suivi) et les facteurs perçus par les médecins comme des obstacles à l´aide au sevrage tabagique. Nous avons appelé tous les médecins sélectionnés à l´échantillonnage, afin d´expliquer le but de l´étude, les encourager à participer, confirmer la confidentialité des réponses et obtenir leur consentement. Des courriels électroniques, avec le lien vers le questionnaire, ont été envoyés aux médecins. Certains éléments du questionnaire ont été tirés d´instruments utilisés dans des études antérieures pour évaluer les connaissances, les attitudes et les pratiques des médecins en matière de sevrage tabagique et ceux-ci ont été modifiés pour convenir à cette étude. En effet, le questionnaire a été formulé en se référant à la mise à jour de 2008 des directives de pratique clinique pour le traitement de l´usage du tabac et de la dépendance par le service américain de santé publique [7]. En outre, les données pour le développement du questionnaire ont été tirées d´un questionnaire sur les pratiques des prestataires de soins de santé en matière d´interventions d´abandon du tabac développé par l´école de santé publique à l´Université de Nairobi au Kenya en collaboration avec l´école de santé publique à l´Université de Makerere en Ouganda [8], d´un instrument de mesure des connaissances, attitudes et pratiques en matière de tabagisme chez les prestataires de soins de santé (S-KAP) développé par des chercheurs à l´Université de Californie de San Francisco [9] et d´un questionnaire sur les habitudes tabagiques, les connaissances, les attitudes et les interventions de lutte antitabac chez les médecins et les infirmières développé par le département de médecine de famille à l´Université de Duzce en Turquie [10].

**Considérations éthiques:** avant de mener l´enquête, il a été nécessaire d´obtenir le consentement éclairé des médecins après leur information sur le déroulement et l´objectif de l´étude. Les données personnelles des médecins n´étaient pas divulguées à une tierce personne et l´exploitation des questionnaires était faite dans l´anonymat.

**Saisie et analyse des données:** la saisie et l´analyse des données ont été faites par le logiciel SPSS. La description des variables qualitatives était réalisée par la détermination des fréquences absolues et relatives. Celle des variables quantitatives était menée par les moyennes ±écart type (ET) lorsqu´elles étaient distribuées normalement ou sous forme de médianes ±intervalle interquartile (IIQ) dans le cas contraire. La normalité a été vérifiée par le test de Kolmogorov-Smirnov.

## Résultats

**Caractéristiques sociodémographiques:** la population étudiée comportait 115 médecins de première ligne dont 60 (52,2%) étaient de sexe féminin, soit un sex-ratio (H/F) de 0,91. L´âge médian était de 43 ans (IIQ=[34-55 ans]). Soixante-sept médecins (66,1%) exerçaient au secteur privé. La médiane des années de pratique était de 10 ans (IIQ=[4-25 ans]). Soixante-dix-huit répondants (67,8%) ont déclaré n´avoir jamais fumé. La prévalence du tabagisme chez notre échantillon de médecins de première ligne était de 22,6% (n=26). La prévalence du tabagisme était de 38,2% (n=21) chez les médecins de sexe masculin et de 8,3% (n=5) chez les médecins de sexe féminin. La durée médiane du tabagisme était de 15 ans (IIQ=[8-20 ans]). La quantité médiane de cigarettes fumées était de 10 cigarettes par jour (IIQ=[5-20 cigarettes]). Parmi les fumeurs actuels, 18 (69,2%) ont déjà essayé d´arrêter de fumer.

**Connaissances et formation des médecins en matière d´aide au sevrage tabagique:** parmi les médecins enquêtés, 86 (74,8%) approuvaient le rôle primordial du médecin de première ligne dans l´aide à l´arrêt du tabagisme. Quatre-vingt-dix-huit répondants (85,2%) n´ont pas eu de formation sur l´accompagnement à l´arrêt du tabagisme en postuniversitaire. Le test de motivation à arrêter le tabac de Lagrue et Légeron était le moins connu (n=21; 18,3%), tandis que la thérapie de substitution nicotinique était la plus connue (n=71; 61,7%) (Tableau 1). Parmi les répondants, 67% (n=77) avaient le sentiment d´être moyennement formés pour aider leurs patients fumeurs à arrêter de fumer, alors que 18,3% (n=21) s´estimaient non formés du tout pour aider au sevrage tabagique. Quatre-vingt pour cent des médecins enquêtés (n= 92) souhaitaient participer à une réunion d´information sous forme de séminaires ou de congrès, tandis que 57,4% (n=66) souhaitaient participer à une formation telle qu´un diplôme de tabacologie.

**Tableau 1:** répartition des médecins selon leurs connaissances concernant les scores et les échelles utilisés dans l´aide au sevrage tabagique et la thérapie de substitution nicotinique

Connaissances des médecins en matière d´aide au sevrage tabagique	Nombre	Pourcentage
**Le test de Fagerström de dépendance à la nicotine**	29	25,2%
**Le test de motivation à arrêter le tabac de Lagrue et Légeron**	21	18,3%
**L´échelle HAD d´évaluation de l'anxiété et de la dépression de Sigmond et Snaith**	26	22,6%
**La thérapie de substitution nicotinique**	71	61,7%

**Attitudes des médecins en matière d´aide au sevrage tabagique:** nous avons noté que 63,5% des répondants (n=73) étaient convaincus qu´il est de la responsabilité des médecins d´aider leurs patients à arrêter de fumer. Par ailleurs, 87% des répondants (n=100) pensaient que les médecins devraient donner le bon exemple en ne fumant pas et 92,2% (n=106) étaient convaincus que les médecins devraient être formés en matière d´aide au sevrage tabagique pour pouvoir convaincre les patients à arrêter de fumer. Cependant, 33,9% des répondants (n=39) estimaient qu´il est inconfortable de conseiller aux patients fumeurs d´arrêter de fumer. D´autre part, 47,8% des répondants (n=55) estimaient qu´ils n´avaient pas suffisamment de temps pour conseiller à tous les patients fumeurs d´arrêter de fumer, tandis que 33% (n=38) ont perçu que les autres problèmes de santé de leurs patients primaient sur les conseils de sevrage tabagique (Tableau 2).

**Tableau 2:** répartition des médecins selon leurs attitudes en matière d´aide au sevrage tabagique

Attitudes des médecins en matière d'aide au sevrage tabagique	Être en désaccord avec l'attitude n (%)	Être en accord avec l'attitude n (%)
C'est la responsabilité du médecin d'aider les patients à arrêter de fumer	42 (36,5)	73 (63,5)
Les conseils d´un médecin aident à motiver les fumeurs à cesser de fumer	10 (8,7)	105 (91,3)
Cesser de fumer est un choix individuel, ce n´est pas le rôle du médecin de conseiller aux patients d'arrêter	83 (72,2)	32 (27,8)
Les médecins devraient donner le bon exemple en ne fumant pas	15 (13)	100 (87)
Les médecins devraient être formés pour conseiller les patients	9 (7,8)	106 (92,2)
Les conseils de sevrage tabagique améliorent la relation médecin-patient	33 (28,7)	82 (71,3)
Il est inconfortable de conseiller aux patients fumeurs d´arrêter	76 (66,1)	39 (33,9)
Le médecin n´a pas suffisamment de temps pour conseiller à tous les patients d´arrêter	60 (52,2)	55 (47,8)
Les autres problèmes de santé des patients sont prioritaires	77 (67)	38 (33)
Il ne vaut pas la peine de discuter des avantages du sevrage avec les patients, car ils savent déjà qu´ils doivent arrêter de fumer	91 (79,1)	24 (20,9)

n: nombre; % :pourcentage

**Pratiques des médecins en matière d´aide au sevrage tabagique:** dans la composante « demander » des 5A, les répondants interrogeaient systématiquement les patients sur leurs habitudes tabagiques en présence de symptômes respiratoires dans 83,5% des cas (n=96) et en présence de maladies liées au tabac dans 87,8% des cas (n=101), mais seulement 39,1% des médecins enquêtés (n=45) le faisaient systématiquement chez tout malade. Globalement, 66% des médecins enquêtés (n=76) effectuaient toujours la composante « demander » des 5A dans leur pratique quotidienne. Dans la composante « conseiller », 66,1% des médecins enquêtés (n=76) ont déclaré qu´ils conseillaient toujours aux patients fumeurs d´arrêter. En outre, 65,2% des répondants (n=75) ont déclaré qu´ils discutaient toujours des méfaits du tabac et des bienfaits du sevrage tabagique avec les patients. Ainsi, globalement, 65% des médecins enquêtés (n=75) effectuaient toujours la composante « conseiller » des 5A dans leur pratique quotidienne. Concernant la composante « évaluer », 52,2% des médecins enquêtés (n=60) ont déclaré qu´ils évaluaient toujours si les patients étaient prêts à arrêter de fumer. Dans la composante « aider », 22,6% des répondants (n=26) aidaient toujours les patients fumeurs à fixer une date d´arrêt et 13% (n=15) discutaient toujours l´utilisation d´une thérapie de substitution nicotinique. Globalement, 14% des médecins enquêtés (n=16) effectuaient toujours la composante « aider » des 5A dans leur pratique quotidienne. Dans la composante « organiser un suivi » des 5A, 17,4% des répondants (n=20) ont déclaré qu´ils organisaient toujours un suivi des patients après avoir cessé de fumer pour évaluer leurs progrès sur l´arrêt du tabac (Tableau 3).

**Tableau 3:** répartition des médecins selon leurs pratiques en matière d´aide au sevrage tabagique

Pratiques des médecins en matière d'aide au sevrage tabagique	Fréquence des pratiques		
	Jamais n (%)	Parfois n (%)	Toujours n (%)
**Demander**			
Interroger les patients sur leurs habitudes tabagiques			
En présence de symptômes respiratoires	0	19 (16,5)	96 (83,5)
En présence d´une maladie liée au tabac	0	14 (12,2)	101 (87,8)
Chez tout malade	7 (6,1)	63 (54,8)	45 (39,1)
Inscrire l´histoire tabagique du patient fumeur dans le dossier médical	3 (2,6)	44 (38,3)	68 (59,1)
Demander le nombre de cigarettes fumées par jour	4 (3,5)	39 (33,9)	72 (62,6)
**Conseiller**			
Conseiller l´arrêt	3 (2,6)	36 (31,3)	76 (66,1)
Parler aux patients fumeurs des méfaits du tabac et des bienfaits du sevrage tabagique	7 (6,1)	33 (28,7)	75 (65,2)
**Evaluer**			
Evaluer la volonté des patients fumeurs à arrêter de fumer	10 (8,7)	45 (39,1)	60 (52,2)
**Aider**			
Aidez les patients fumeurs à fixer une date d´arrêt	42 (36,5)	47 (40,9)	26 (22,6)
Donner des brochures d´aide à l´arrêt aux patients fumeurs	79 (68,7)	28 (24,3)	8 (7)
Discuter l´utilisation d´une thérapie de substitution nicotinique	41 (35,7)	59 (51,3)	15 (13)
**Organiser un suivi**			
Organiser un suivi des patients fumeurs après avoir cessé de fumer	58 (50,4)	37 (32,2)	20 (17,4)

n: nombre; %: pourcentage

**Obstacles rencontrés dans l´aide au sevrage tabagique:** le désintérêt des patients à recevoir des informations sur l´arrêt du tabac était jugé, par 53% des médecins enquêtés (n=61), comme obstacle important pour pratiquer l´aide au sevrage tabagique. L´absence de matériel éducatif était jugée comme obstacle important par 27% des répondants (n=31) et l´absence de formation suffisante était jugée comme obstacle important par 25,2% des répondants (n=29).

## Discussion

Dans la présente étude, la prévalence du tabagisme chez les médecins de première ligne enquêtés était de 22,6%. Cette prévalence était proche de celle retrouvée chez la population générale en Tunisie en 2018 qui était de 26% avec une prévalence de 49,1% chez les hommes et 2,9% chez les femmes [2]. De même, la prévalence du tabagisme retrouvée chez notre échantillon était proche de la prévalence du tabagisme chez un échantillon de médecins et de pharmaciens de l´hôpital Charles Nicolles de Tunis en 2005 [11]. En revanche, elle était supérieure à la prévalence du tabagisme retrouvée chez un échantillon de médecins de l´hôpital Farhat Hached de Sousse en 2008 [4]. Par ailleurs, des études menées dans de nombreux pays africains ont révélé des prévalences de tabagisme chez les médecins qui variaient de 3% au Nigeria et 3,6% au Cameroun jusqu´à 16,3% au Maroc [6, 12-14]. Ainsi, les prévalences du tabagisme chez les médecins dans les différents pays africains étaient inférieures à la prévalence du tabagisme chez les médecins dans notre étude. En comparant nos résultats aux résultats d´études menées dans des pays du Moyen-Orient, la prévalence du tabagisme dans notre échantillon de médecins de première ligne était plus élevée que celles retrouvées à Bahreïn [15] et en Arabie Saoudite [16, 17]. En outre, par rapport aux pays européens, la prévalence du tabagisme chez notre échantillon de médecins était proche de celle retrouvée en Italie [18], tandis qu´elle était inférieure à la prévalence notée en Roumanie [19] et supérieure à celle notée en Espagne [20]. Par ailleurs, à l´échelle mondiale, une revue de la littérature avec une méta-analyse [21] a révélé que, parmi tous les pays à revenu faible ou intermédiaire, les prévalences de tabagisme chez les personnels de santé les plus élevées (> 50%) étaient retrouvées en Tunisie et au Pakistan, tandis que la prévalence la plus faible (<10%) était notée en Inde.

Bien que les trois quarts des médecins participants à cette étude étaient conscients du rôle primordial des médecins de première ligne dans l´aide au sevrage tabagique, seulement la moitié ont reçu une formation médicale continue sur l´accompagnement à l´arrêt du tabagisme. Ces constations étaient concordantes avec la littérature. En effet, des études menées en Chine, en Arabie Saoudite et en Égypte, ont révélé qu´uniquement la moitié des médecins de première ligne ont reçu une formation sur l´aide au sevrage tabagique [6, 22, 23]. En outre, dans des études menées en Bosnie et en Australie, moins de la moitié des professionnels de la santé ont déclaré avoir reçu une formation sur les stratégies de l´aide au sevrage tabagique [24, 25]. Par ailleurs, dans une étude menée au Kenya, 89% des médecins enquêtés ont déclaré n´avoir reçu aucune formation sur l´aide au sevrage tabagique [8]. Des connaissances insuffisantes des divers tests et échelles utilisés dans l´aide à l´arrêt du tabac pourrait entraver la prestation de cette intervention aux patients. Dans notre étude, les trois quarts des médecins enquêtés n´avaient pas une idée sur le test de Fagerström de dépendance à la nicotine, le test de motivation à arrêter le tabac de Lagrue et Légeron ou l´échelle HAD d´évaluation de l´anxiété et de la dépression de Sigmond et Snaith. Ce résultat a été également constaté dans une étude menée en Chine où le un-quart des médecins enquêtés ont déclaré ne pas avoir de connaissances suffisantes pour l´aide au sevrage tabagique [22]. Par ailleurs, les deux tiers des participants à cette étude avaient une idée sur la thérapie de substitution nicotinique. Ceci pourrait être expliqué par l´engagement de la Tunisie dans un processus de lutte anti-tabac depuis les années 1980 [26], avec l´adoption de stratégies validées au niveau mondial, notamment la CCLAT [27], qui avait pour axes stratégiques la formation approfondie, théorique et pratique des médecins de première ligne en matière de lutte contre le tabagisme et la généralisation des consultations de sevrage tabagique dans toutes les régions [26].

Toutefois, malgré la ratification de la CCLAT en 2010 et l´instauration de la stratégie nationale de lutte contre le tabagisme [28], nous n´avons pas observé l´impact de cette stratégie sur les compétences des médecins de première ligne, ni sur la généralisation des consultations de sevrage tabagique. En comparaison à nos résultats, dans une étude menée auprès de médecins de première ligne en Chine, seulement la moitié des personnes interrogées connaissaient la thérapie de substitution nicotinique [22]. Le manque de connaissance de la thérapie de substitution nicotinique utilisée pour aider les fumeurs à faire face aux symptômes de sevrage tabagique, pourrait limiter la capacité des médecins de discuter efficacement de ces options de traitement avec les fumeurs. Notre étude a aussi objectivé que 14,8% des médecins interrogés estimaient qu´ils étaient bien formés pour aider leurs patients fumeurs à arrêter de fumer. De même, une étude menée en Arabie Saoudite a révélé que 29,2% des médecins étaient satisfaits de leurs compétences en matière de sevrage tabagique à leurs patients fumeurs [17]. En revanche, dans une étude menée en Bosnie, environ les deux tiers des professionnels de la santé se sentaient vraiment ou assez préparés à aider leurs patients fumeurs à arrêter de fumer [25]. En outre, dans notre étude, la plupart des médecins enquêtés ont déclaré qu´ils aimeraient avoir une formation sur l´aide au sevrage tabagique. Cela concordait avec les résultats d´une étude menée en Australie dans laquelle 71% des professionnels de la santé souhaitaient participer à une formation sur l´aide au sevrage tabagique [24]. De même, une étude menée au Kenya a révélé que 96% des médecins étaient prêts à recevoir une formation sur l´aide au sevrage tabagique [8].

Dans notre étude, la majorité des médecins enquêtés avaient une attitude positive à l´égard de l´aide au sevrage tabagique. Ce résultat était concordant avec les résultats de recherches antérieures [6, 17, 23]. Notre étude a objectivé que les deux tiers des médecins enquêtés considéraient que c´est la responsabilité du médecin d´aider les patients fumeurs à arrêter de fumer. Ceci a été également observé auprès d´un échantillon de médecins chinois, dont les trois quarts ont déclaré que les médecins devraient aider les fumeurs à cesser de fumer [22]. Par ailleurs, dans une étude menée en Arabie Saoudite auprès d´un échantillon de médecins de première ligne, tous les répondants considéraient que l´aide au sevrage tabagique faisait partie de leurs tâches [23]. De plus, dans notre étude, 92,2% des répondants considéraient que les médecins devraient être formés en sevrage tabagique pour pouvoir aider les patients fumeurs à arrêter de fumer. De même, dans une étude menée en Arabie Saoudite, environ les deux tiers des médecins enquêtés déclaraient que les conseils de sevrage tabagique nécessitent une formation spéciale [23]. En outre, les deux tiers des médecins participants à notre étude ont déclaré qu´ils ne se sentaient pas gênés à donner des conseils de sevrage tabagique aux patients fumeurs. Cela concordait avec les résultats d´une étude menée en Arabie Saoudite dans laquelle les trois quarts des médecins de première ligne ont déclaré que ce n´était pas inconfortable de donner de tels conseils [23].

Dans la présente étude, les différentes composantes du modèle 5A des pratiques n´étaient pas exécutées à la même fréquence. En effet, les pourcentages les plus élevés pour les cinq sous-éléments du modèle 5A ont été obtenus pour les composantes « Demander » (66%) et « Conseiller » (65%), suivies par la composante « Évaluer » (52,2%). Tandis que les pourcentages les plus bas ont été obtenus pour les composantes « Aider » (14%) et « Organiser un suivi » (17,4%). Ces constations étaient concordantes avec la littérature. En effet, des recherches antérieures [6, 8, 29-33] ont suggéré que les activités les moins exécutées de la stratégie 5A étaient les composantes « Aider » et « Organiser un suivi ».

Notre étude a aussi objectivé que plus que 80% des médecins enquêtés interrogeaient les patients sur leurs habitudes tabagiques en présence de symptômes respiratoires ou de maladie liée au tabac, alors que 39,1% seulement interrogeaient systématiquement tous les patients sur leurs habitudes tabagiques. Cependant, la proportion de médecins qui demandaient systématiquement le statut tabagique de leurs patients était égale à 60% dans la population de l´étude choisie pour déterminer le nombre minimal de sujets nécessaires dans notre étude [6], en se basant sur cette proportion comme variable d´intérêt principal. Cet écart a été observé bien que nous avons choisi une population de médecins de première ligne égyptiens, qui était la plus comparable à notre population en termes de statut socio-économique de la zone d´étude par rapport à d´autres études sur les interventions d´aide à l´arrêt du tabac. De même, d´autres études [20, 22] ont trouvé que plus que la moitié des médecins enquêtés interrogeaient systématiquement leurs patients sur leurs habitudes tabagiques. Néanmoins, des études réalisées au Kenya, en Turquie et en Australie [8, 10, 24] ont trouvé que seulement le un tiers des médecins enquêtés demandaient à tous leurs patients s´ils fumaient.

En outre, 52,1% des médecins participants à notre étude inscrivaient l´histoire tabagique de leurs patients fumeurs dans le dossier médical et 62,6% demandaient le nombre de cigarettes fumées par jour. En revanche, dans une étude menée au Kenya, uniquement 29% du personnel de santé enregistraient le statut tabagique des patients dans le dossier médical et 38,5% demandaient le nombre de cigarettes fumées [8]. De plus, les deux tiers des médecins enquêtés dans notre étude conseillaient l´arrêt à tous les patients fumeurs. Ceci a été également observé auprès d´un échantillon de médecins chinois, dont 79% conseillaient toujours l´arrêt à leurs patients fumeurs [22]. Cependant, des études menées au Kenya et en Australie [8, 24] ont trouvé que les médecins conseillaient l´arrêt du tabac aux patients fumeurs dans respectivement 43,5% et 19% des cas. Par ailleurs, dans notre étude, les deux tiers des médecins parlaient aux patients fumeurs des méfaits du tabac et des bienfaits du sevrage tabagique. Dans des études menées en Chine et au Kenya [8, 22], seulement 43,6% et 29,3% des médecins discutaient des risques et des avantages du sevrage tabagiques avec leurs patients fumeurs. Dans notre étude, environ le un-quart des répondants aidaient toujours les patients fumeurs à fixer une date d´arrêt. Cela concordait avec les résultats d´une étude menée en Chine [22]. Néanmoins, d´autres études ont trouvé que plus que la moitié des médecins enquêtés aidaient leurs patients fumeurs à fixer une date d´arrêt [30, 33]. Par ailleurs, nos résultats ont révélé que seulement 13% des médecins enquêtés discutaient systématiquement l´utilisation d´une thérapie de substitution nicotinique avec leurs patients fumeurs. Ces constations étaient concordantes avec les résultats de recherches antérieures [22, 30].

La présente étude a montré que le manque d´intérêt des patients était jugé, par la moitié des médecins enquêtés, comme obstacle important pour mener des interventions d´aide au sevrage tabagique. Une étude menée en Égypte a trouvé que le manque de formation était l´obstacle le plus important lié au médecin, tandis que celui lié au patient était son désir d´arrêter de fumer [6]. Une autre étude menée aux États Unis a révélé que le manque de temps, le manque de formation des médecins et le manque d´intérêt des patients étaient des obstacles importants dans l´aide au sevrage tabagique [29]. En outre, le personnel soignant interrogé dans une étude menée au Kenya a déclaré que la formation insuffisante et l´absence de directives claires en matière d´aide au sevrage tabagique constituaient des obstacles majeurs empêchant les répondants d´intervenir auprès des patients fumeurs [8]. Néanmoins, des médecins de première ligne enquêtés dans des études menées en Turquie et en Arabie Saoudite ont déclaré que le manque de temps était l´obstacle principal dans l´aide au sevrage tabagique [10, 30].

**Limites:** la présente étude reposait principalement sur les auto-déclarations des médecins, qui pourraient ainsi avoir tendance à surévaluer leurs prestations d´aide au sevrage tabagique. Il aurait pu être plus intéressant d´assister à une consultation avec le médecin et de juger objectivement sa prestation. La mise en œuvre d´une étude nationale avec un échantillon plus large et une population de participants plus diversifiée est recommandée.

## Conclusion

Nos résultats ont révélé que la plupart des médecins de première ligne interrogés avaient besoin de plus de formation en matière d´aide au sevrage tabagique. Ceci doit inciter à mettre en place un enseignement de la pathologie du tabac et à intégrer dans le champ médical la prévention et l´éducation contre le tabagisme. En effet, l´éducation des médecins sur les tests simples et les différents traitements utilisés dans l´aide au sevrage tabagique, aiderait à accroître leur confiance et leurs compétences pour réussir à aider les patients fumeurs à arrêter de fumer [24]. Ainsi, l´évaluation et l´amélioration des directives nationales pour le dépistage, la documentation et le traitement de la dépendance au tabac s´avèrent nécessaires.

### Etat des connaissances sur le sujet

- L´épidémie de tabagisme constitue une menace pour la santé publique dans le monde et en Tunisie;
- Le médecin de première ligne joue un rôle primordial dans l´aide au sevrage tabagique.

### Contribution de notre étude à la connaissance

- La prévalence du tabagisme chez les médecins de première ligne du gouvernorat de Sfax était supérieure à celle retrouvée dans des pays africains, des pays du Moyen-Orient et des pays européens;
- La plupart des médecins de première ligne du gouvernorat de Sfax nécessitent plus de formation en matière d´aide au sevrage tabagique.

## References

[1] Organisation mondiale de la Santé (OMS). Tabac.

[2] World Health Organization (WHO) WHO global report on trends in prevalence of tobacco use 2000-2025: third edition.

[3] Fakhfakh R, Mohamed H, Maalej M, Achour N, Nacef T (2002). Tabagisme en Tunisie: comportements et connaissances. Bulletin de l´Organisation mondiale de la Santé: recueil d´articles.

[4] Ben Salah SM, Rhif H, Elguesmi O, Ben Abderrahmen A, Hayouni A, Mrizak N (2011). Connaissances, attitudes et comportements du personnel hospitalier vis-à-vis du tabagisme et de la réglementation anti-tabac: résultats d´une enquête réalisée au CHU F-Hached de Sousse (Tunisie). Rev Pneumol Clin.

[5] Zhu S, Melcer T, Sun J, Rosbrook B, Pierce JP (2000). Smoking cessation with and without assistance: a population-based analysis. Am J Prev Med.

[6] Eldein HN, Mansour NM, Mohamed SF (2013). Knowledge, Attitude and Practice of Family Physicians Regarding Smoking Cessation Counseling in Family Practice Centers, Suez Canal University, Egypt. J Family Med Prim Care.

[7] The Clinical Practice Guideline Treating Tobacco Use and Dependence 2008 Update Panel L (2008). A Clinical Practice Guideline for Treating Tobacco Use and Dependence: 2008 Update: A U.S. Public Health Service Report. Am J Prev Med.

[8] Gichuki JW, Opiyo R, Mugyenyi P, Namusisi K (2015). Healthcare Providers´ Level of Involvement in Provision of Smoking Cessation Interventions in Public Health Facilities in Kenya. J Public Health Afr.

[9] Delucchi KL, Tajima B, Guydish J (2009). Development of the Smoking Knowledge, Attitudes, and Practices (S-KAP) Instrument. J Drug Issues.

[10] Sonmez CI, Aydin LY, Turker Y, Baltaci D, Dikici S, Sariguzel YC (2015). Comparison of smoking habits, knowledge, attitudes and tobacco control interventions between primary care physicians and nurses. Tob Induc Dis.

[11] Fakhfakh R, Khanchal F, Klouz A, Salah FB, Lakhal M, Belkahia C (2011). Determinants of tobacco use habits among hospital staff in Tunisia, 2005. Prev Med.

[12] Nollen NL, Adewale S, Okuyemi KS, Ahluwalia JS, Parakoyi A (2004). Workplace tobacco policies and smoking cessation practices of physicians. J Natl Med Assoc.

[13] Mbatchou Ngahane BH, Luma H, Ndiaye M, Njankouo YM, Mbahe S, Wandji A (2012). Prévalence du tabagisme chez le personnel de l´Hôpital Général de Douala, Cameroun. Pan Afr Med J.

[14] Badri F, Sajiai H, Amro L (2017). Prévalence du tabagisme chez le personnel médical et paramédical du CHU Mohamed VI à Marrakech. Pan Afr Med J.

[15] Borgan SM, Jassim G, Marhoon ZA, Almuqamam MA, Ebrahim MA, Soliman PA (2014). Prevalence of tobacco smoking among health-care physicians in Bahrain. BMC Public Health.

[16] Al-Hagabani MA, Khan MS, Al-Hazmi AM, Shaher BM, El-Fahel AO (2020). Smoking behavior of primary care physicians and its effect on their smoking counseling practice. J Fam Med Prim Care.

[17] Al-Jdani S, Mashabi S, Alsaywid B, Zahrani A (2018). Smoking cessation counseling: Knowledge, attitude and practices of primary healthcare providers at National Guard Primary Healthcare Centers, Western Region, Saudi Arabia. J Fam Community Med.

[18] Incorvaia C, Pravettoni C, Dugnani N, Riario-Sforza GG (2008). A survey on current attitudes to smoking in health care workers in Italy. Med Lav.

[19] Poantă LI, Zdrenghea D, Albu A (2006). Psychometric evaluation of Romanian version of Job Content Questionnaire in physicians. Rom J Intern Med.

[20] Jiménez-Ruiz CA, Riesco Miranda JA, Ramos Pinedo A, de Higes Martinez E, Marquez FL, Palomo Cobos L (2015). Prevalence of and Attitudes towards Smoking among Spanish Health Professionals. Respir Int Rev Thorac Dis.

[21] Nilan K, McKeever TM, McNeill A, Raw M, Murray RL (2019). Prevalence of tobacco use in healthcare workers: A systematic review and meta-analysis. PLoS One.

[22] Klink K, Lin S, Elkin Z, Strigenz D, Liu S (2011). Smoking cessation knowledge, attitudes, and practice among community health providers in China. Fam Med.

[23] AlAteeq M, Alrashoud AM, Khair M, Salam M (2016). Smoking cessation advice: the self-reported attitudes and practice of primary health care physicians in a military community, central Saudi Arabia. Patient Prefer Adherence.

[24] Pilkington A, Carter O, Cameron A, Thompson S (2009). Tobacco control practices among Aboriginal health professionals in Western Australia. Aust J Prim Health.

[25] Hodgetts G, Broers T, Godwin M (2004). Smoking behaviour, knowledge and attitudes among Family Medicine physicians and nurses in Bosnia and Herzegovina. BMC Fam Pract.

[26] Latiri H, Aissa S, Ben Rejeb M, Chebil D, Dhidah L (2015). La consultation d´aide au sevrage tabagique du CHU Sahloul: résultats du suivi et facteurs prédictifs d´échec. Rev Tunis Santé Publique.

[27] Organisation mondiale de la Santé (OMS) Convention-cadre de l´OMS pour la lutte antitabac.

[28] Ministère de la santé publique Textes juridiques et règlementaires. Prévention des méfaits du tabagisme.

[29] Soto Mas FG, Papenfuss RL, Jacobson HE, Hsu CE, Urrutia-Rojas X, Kane WM (2005). Hispanic physicians´ tobacco intervention practices: a cross-sectional survey study. BMC Public Health.

[30] Jradi H (2017). Awareness, practices, and barriers regarding smoking cessation treatment among physicians in Saudi Arabia. J Addict Dis.

[31] Kruger J, O´Halloran A, Rosenthal A (2015). Assessment of compliance with US Public Health Service Clinical Practice Guideline for tobacco by primary care physicians. Harm Reduct J.

[32] Bartsch A-L, Härter M, Niedrich J, Brütt AL, Buchholz A (2016). A Systematic Literature Review of Self-Reported Smoking Cessation Counseling by Primary Care Physicians. PLoS One.

[33] Tong EK, Strouse R, Hall J, Kovac M, Schroeder SA (2010). National survey of U.S. health professionals´ smoking prevalence, cessation practices, and beliefs. Nicotine Tob Res.

